# Neonatal outcomes in pregnancies complicated by placenta accreta- a matched cohort study

**DOI:** 10.1007/s00404-023-07353-6

**Published:** 2024-01-23

**Authors:** Shlomi Toussia-Cohen, Elias Castel, Lior Friedrich, Nizan Mor, Aviran Ohayon, Gabriel Levin, Raanan Meyer

**Affiliations:** 1 The Department of Obstetrics and Gynecology, Chaim Sheba Medical Center, Tel-Hashomer, Ramat-Gan, Israel; 2 The Sackler Faculty of Medicine, Tel-Aviv University, Tel-Aviv, Israel; 3 The Joyce & Irving Goldman Medical School, Faculty of Health Sciences, Ben-Gurion University of the Negev, Beer Sheva, Israel; 4 The Department of Gynecologic Oncology, Hadassah Medical Center, Jerusalem, Israel

**Keywords:** Placenta accreta spectrum, Cesarean delivery, Neonatal outcome, Composite adverse neonatal outcome, Neonatal intensive care unit

## Abstract

**Purpose:**

Pregnancies complicated by placenta accreta spectrum (PAS) are associated with severe maternal morbidities. The aim of this study is to describe the neonatal outcomes in pregnancies complicated with PAS compared with pregnancies not complicated by PAS.

**Methods:**

A retrospective cohort study conducted at a single tertiary center between 03/2011 and 01/2022, comparing women with PAS who underwent cesarean delivery (CD) to a matched control group of women without PAS who underwent CD. We evaluated the following adverse neonatal outcomes: umbilical artery pH < 7.0, umbilical artery base excess ≤ − 12, APGAR score < 7 at 5 min, neonatal intensive care unit (NICU) admission, mechanical ventilation, hypoxic ischemic encephalopathy, seizures and neonatal death. We also evaluated a composite adverse neonatal outcome, defined as the occurrence of at least one of the adverse neonatal outcomes described above. Multivariable regression analysis was used to determine which adverse neonatal outcome were independently associated with the presence of PAS.

**Results:**

265 women with PAS were included in the study group and were matched to 1382 controls. In the PAS group compared with controls, the rate of composite adverse neonatal outcomes was significantly higher (33.6% vs. 18.7%, respectively, *p* < 0.001). In a multivariable logistic regression analysis, Apgar score < 7 at 5 min, NICU admission and composite adverse neonatal outcome were independently associated with PAS.

**Conclusion:**

Neonates in PAS pregnancies had higher rates of adverse outcomes. Apgar score < 7 at 5 min, NICU admission and composite adverse neonatal outcome were independently associated with PAS.

**Supplementary Information:**

The online version contains supplementary material available at 10.1007/s00404-023-07353-6.

## What does this study add to the clinical work

**Table:** 

Neonates in placenta accreta spectrum pregnancies have higher rates of adverse neonatal outcomes. Low Apgar score, NICU admission and composite adverse neonatal outcome were independently associated with placenta accreta spectrum.	

## Introduction

Placenta accreta spectrum (PAS) is the pathologic placental adherence to the myometrium, most commonly hypothesized as due to a defect in the uterine endometrial-myometrial interface leading to abnormal trophoblast invasion of the myometrium [1]. PAS includes placenta accreta, placenta increta and placenta percreta [2, 3]. The incidence of PAS is estimated to be as high as 1.1% of all births [4], and is rising globally due to an increase in the prevalence of the established risk factors, mainly cesarean deliveries (CD) and also other uterine surgeries such as surgical uterine evacuation, myomectomy and infertility treatments [5–8].

The most common surgical approach for management of PAS pregnancies is cesarean hysterectomy or peripartum hysterectomy [4]. In recent years, mostly for women interested in future fertility, uterine preserving PAS surgery has been increasingly performed [9, 10].

Pregnancies complicated by PAS are associated with severe maternal morbidities such as life threatening hemorrhage, and damage to adjacent organs, mainly urinary tract and gastrointestinal tract [11]. While maternal outcomes following pregnancies complicated by PAS are well reported and described, reports regarding neonatal outcomes following these pregnancies are scarce. CD is a well-established risk factor for adverse neonatal outcomes compared with vaginal delivery [12], thus it is reasonable that a complicated CD due to PAS may further impact neonatal outcomes. Previous retrospective studies most consistently reported high rates of neonatal intensive care unit (NICU) admissions and a relatively high need for mechanical ventilation in PAS pregnancies [13–16]. These studies reported only a limited number of neonatal outcomes and did not match them to non-PAS pregnancies.

The aim of this study is to evaluate the association between PAS and adverse neonatal outcomes.

## Materials and methods

This was a retrospective matched cohort study conducted at a single tertiary center. Historical data via electronic medical records of pregnant women diagnosed with PAS who underwent CD between 03/2011 and 01/2022 were reviewed. Data were originally collected prospectively including umbilical artery pH and base excess for all women. Women diagnosed with PAS undergoing CD were matched to a control group of women not diagnosed with PAS undergoing a repeat CD with a 1:6 ratio. Vaginal deliveries were excluded. Matching was performed for gestational age at CD and the number of prior CDs.

We excluded pregnancies with intrauterine fetal death, multiple gestations and congenital anomalies.

We evaluated the following adverse neonatal outcomes: umbilical artery pH < 7.0, umbilical artery base excess ≤ − 12, APGAR score < 7 at 5 min, neonatal intensive care unit (NICU) admission, mechanical ventilation, hypoxic ischemic encephalopathy, seizures and neonatal death. We also evaluated a composite adverse neonatal outcome, defined as the occurrence of at least one of the adverse neonatal outcomes described above.

Maternal baseline characteristics included age, body mass index, smoking, diabetes mellitus, hypertensive disease, time interval since last CD, number of previous CDs, in vitro fertilization and emergent CD.

PAS was defined in accordance with the ‘International Federation of Gynecology and Obstetrics’ classification for the clinical diagnosis of placenta accreta spectrum disorders [17].

Hypertensive Disorders of Pregnancy (HDP) were defined as the presence gestational hypertension or pre-eclampsia according to the ACOG most recent practice bulletin [18]. Gestational diabetes mellitus (GDM) was defined according to the values proposed by Carpenter and Coustan [19]. Smoking was defined as smoking at least one cigarette (or equivalent) per day. Intrauterine growth restriction was defined as birthweight percentile of ≤ 10% according to local charts [20]. Hypoxic ischemic encephalopathy was defined in accordance with international guidelines and current literature [21, 22].

### PAS management protocol

Sonographic findings suggestive of PAS include irregular placental lacunae, loss of the hypoechoic retroplacental-myometrial clear zone, presence of bridging vessels between the placenta and the bladder wall and interruption of the bladder–uterine interface [23]. All women referred to our tertiary center with sonographic or clinical suspicion for PAS are routinely evaluated by an expert sonographer. Once sonographic PAS diagnosis is established, women are further evaluated by a multidisciplinary team that includes an obstetrical PAS specializing surgeon, a gynecologist, an expert sonographer and an obstetric anesthesiologist.

### Uterine preserving procedure

The uterine-preserving procedure is a surgical option for women interested in fertility preservation or who may be at risk of significant bleeding or damage to other organs during a hysterectomy. Following general anesthesia, a preoperative ureteric stent is placed. During the procedure, the surgeons carefully assess the external surface of the uterus for signs of invasion and placental infiltration. The surgeons dissect the bladder from the uterus and ligate the vessels between the uterus to surrounding tissue. Next, an incision is made in the upper segment of the uterus, avoiding the superior edge of the placenta, and the fetus is delivered. The attached placenta is carefully removed, while any large, inseparable sections of retained placenta are removed in en-bloc. The uterine wall is then sutured and reconstructed. To control bleeding, the surgeons may use uterotonic drugs, surgical vessel ligation, an intrauterine balloon tamponade or uterine compression sutures. In cases where there is a high risk of major bleeding, an interventional radiologist may perform a temporary bilateral occlusion of the uterine arteries via internal iliac artery catheterization to reduce bleeding if needed [24].

In some cases, severe intraoperative bleeding precludes uterine preservation. In these cases, after delivery of the fetus, the placenta is left in place, the uterus is immediately sutured, and a hysterectomy is completed.

### Statistical analysis

Normality of the data was assessed using the Shapiro–Wilk or Kolmogorov–Smirnov tests. We compared study groups using Mann–Whitney U test. The chi-square and Fisher’s exact tests were used for comparison of categorical variables. A comparison of PAS versus non-PAS was performed. Multivariable regression analysis was used to determine which adverse neonatal outcomes were independently associated with the presence of PAS. Variables reaching a p < 0.05 in the univariate analysis were included in the multivariable regression analysis. Statistical analyses were conducted using the IBM Statistical Package for the Social Sciences (IBM SPSS v.29; IBM Corporation Inc, Armonk, NY, USA).

## Results

**Fig. 1:**
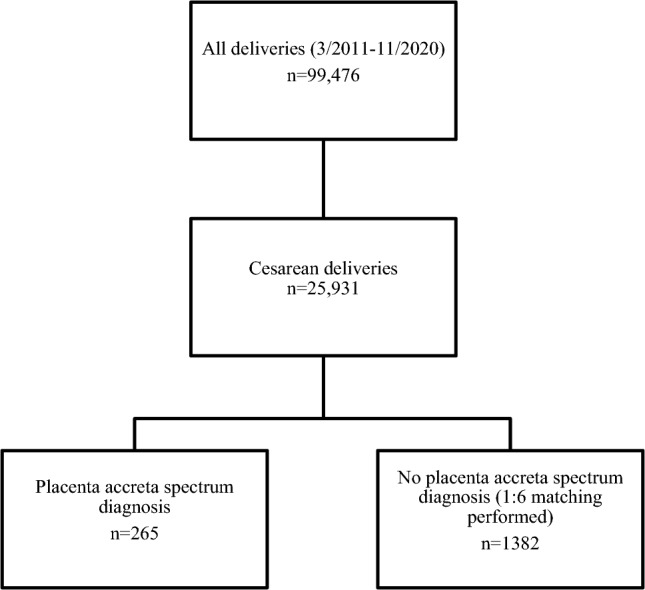
Study population

In the study period, there were 99,476 deliveries, 25,931)26.1%) of which were CDs. Out of these, 265 women PAS met inclusion criteria and were matched to a control group of 1,382 women (Fig. 1).

Maternal baseline characteristics are presented in Table 1. Women with PAS pregnancies, compared to women with non-PAS pregnancies, had lower rates of hypertensive disease (3.4% vs 6.9%, respectively, p = 0.031), underwent more CDs (2.0 vs. 2.0, respectively, p = 0.013) and underwent less emergent CDs (17.8% vs. 42.3%, respectively, p < 0.001) and had higher rates of general anesthesia in the current CD (73.2% vs. 16.3%, respectively, p < 0.001). No other characteristics differed significantly between the two groups. Indications for CD are presented in Table S1.

**Table 1:** Maternal and obstetric baseline characteristics in pregnancies with placenta accreta compared to pregnancies without placenta accreta

Characteristic	Placenta accreta spectrum (n = 265)	No placenta accreta spectrum (n = 1382)	p value
Age, years	35.0 [32.0–39.0]	35.0 [31.0–38.0]	0.068
Body mass index, mean, kg/m2	28.9]25.7–32.0[	28.3]25.4–32.0[	0.416
Smoking	10 (3.9)	72 (5.5)	0.286
Diabetes mellitus	48 (18.1)	237 (17.1)	0.704
Hypertensive disease	9 (3.4)	96 (6.9)	0.031
Preeclampsia	3 (1.1)	38 (2.7)	0.136
Last cesarean delivery, years	3.0 [2.0–5.0]	4.0 [2.0–6.0]	0.075
Number of previous cesarean deliveries	2.0 [1.0–3.0]	2.0 [1.0–3.0]	0.013
In vitro fertilization	20 (7.5)	135 (9.8)	0.257
Emergent cesarean delivery	46 (17.8)	491 (42.3)	< 0.001
General anesthesia	194 (73.2)	225 (16.3)	< 0.001

Data are given as median [Interquartile range] or n (%)

Neonatal outcomes are presented in Table 2. For women with PAS pregnancies, compared to women with non-PAS pregnancies, gestational age at delivery (36^1^ vs. 36^0^ weeks, respectively, p = 0.001) was higher, birthweight (2660.0 g vs. 2625.0, respectively, p = 0.006) was lower. Umbilical artery pH (7.26 vs. 7.28, respectively, p < 0.001) was lower, while the incidence of APGAR score < 7 at 5 min (8.3% vs. 1.0%, respectively, p < 0.001), length of hospital stay (7.0 days vs. 6.0, respectively, p < 0.001), NICU admission (29.8% vs. 18.2%, respectively, p < 0.001), mechanical ventilation (9.8% vs. 3.8%, respectively, p < 0.001) and the neonatal composite score (33.6% vs. 18.7%, respectively, p < 0.001) were significantly higher. No other parameters differed significantly between the two groups.

**Table 2:** Neonatal Outcomes in pregnancies with placenta accreta compared to pregnancies without placenta accreta

Characteristic	Placenta accreta spectrum (n = 265)	No placenta accreta spectrum (n = 1382)	p value
Gestational age at cesarean delivery, weeks days	36^1^ [35^1^–36^4^]	36^0^ [35^1^–36^4^]	0.001
Birthweight, grams	2660.0 [2431.0–2895.0]	2625 [2255.0–2970.0]	0.006
Birthweight percentile	52.0 [33.0, 70.0]	51.0 [29.25, 71.0]	0.788
Intrauterine growth restriction	14 (5.3)	104 (7.5)	0.241
Umbilical artery pH	7.26 [7.22–7.29]	7.28 [7.24–7.31]	< 0.001
Umbilical artery base excess, mEq/L	− 3.1 [− 4.2 to 2.2]	− 3.2 [− 4.6 to − 2.0]	0.334
Umbilical artery pH < 7.0	0 (0.0)	3 (0.2)	> 0.999
Umbilical artery base excess ≤ − 12	0 (0.0)	3 (0.2)	> 0.999
APGAR 5 min < 7	22 (8.3)	14 (1.0)	< 0.001
Hospital stay, days	7.0 [5.5–11.0]	6.0 [5.0–11.0]	< 0.001
Neonatal intensive care unit admission	79 (29.8)	251 (18.2)	< 0.001
Mechanical ventilation	26 (9.8)	53 (3.8)	< 0.001
Hypoxic ischemic encephalopathy	0 (0.0)	3 (0.2)	> 0.999
Seizures	0 (0.0)	5 (0.4)	> 0.999
Neonatal death	1 (0.4)	5 (0.4)	> 0.999
Neonatal composite	89 (33.6)	258 (18.7)	< 0.001

Data are given as median [Interquartile range] or n (%)

Adjusted odds ratio for the risk of adverse neonatal outcomes among patients with PAS compared to controls is presented in Table 3. The following variables were independently associated with PAS: Apgar < 7 at 5 min [adjusted odds ratio (aOR) 95% confidence interval (CI) 2.21 (1.07–4.56), p = 0.031], NICU admission (aOR 95% CI 1.84 (1.05–3.24), p = 0.033) and composite adverse neonatal outcome (aOR 95% CI 2.38 (1.40–4.05), p = 0.001).

**Table 3:** Adjusted odds ratio for the risk of adverse neonatal outcomes among patients with placenta accreta spectrum compared to controls

Characteristic	Adjusted odds ratio	95% Confidence interval	p value
APGAR 5 min < 7^a^	2.21	1.07–4.56	0.031
Neonatal intensive care unit admission^b^	1.84	1.05–3.24	0.033
Mechanical ventilation^c^	1.58	0.77–3.24	0.213
Composite adverse neonatal outcome^d^	2.38	1.40–4.05	0.001

^a^Adjusted for gestational age at delivery, general anesthesia and birthweight

^b^Adjusted for hypertensive disease, gestational age at delivery, emergent cesarean delivery, general anesthesia and birthweight

^c^Adjusted for gestational age at delivery, emergent cesarean delivery, general anesthesia and birthweight

and general anesthesia

^d^Adjusted for gestational age at delivery, birthweight, hypertension, emergent cesarean delivery and general anesthesia

## Discussion

The aim of this study is to describe the neonatal outcomes in pregnancies complicated with PAS compared with pregnancies not complicated by PAS.

We found that Apgar scores below 7, NICU admissions and composite adverse neonatal outcome were independent parameters positively associated with adverse neonatal outcomes.

The occurrence of adverse neonatal outcomes was significantly higher in the PAS group compared to the non-PAS group. Several previous studies described neonatal outcomes among women undergoing surgery due to PAS. These include gestational age at delivery, birthweight, Apgar scores, rates of NICU admissions and need for neonatal mechanical ventilation [13, 15]. Kasraeian et al. [13] described neonatal outcomes in 198 cesarean hysterectomy cases in PAS. Mean gestational age at delivery was at 34 weeks, mean birthweight was 2213 g, Apgar scores in the first and fifth minute were above 6 for most of the neonates. Fifty-seven percent of the neonates were admitted to the NICU. Palacios–Jaraquemada et al. [15] described neonatal outcomes in 315 PAS pregnancies undergoing uterine preserving CD. 5-min Apgar scores were high, admission to the NICU in 81–100% and mechanical ventilation in 2–25% in all cases, rates were higher with higher degree of placental invasion. Eshkoli et al. [16] reviewed 139 PAS-pregnancies and did not find increased risk for adverse perinatal outcomes such as low Apgar scores at 1 and 5 min and perinatal mortality. Fishel Bartal et al. [25] reviewed 109 PAS pregnancies and reported composite neonatal morbidity (Apgar’s score < 5 at 5 min, mechanical ventilation, or respiratory distress syndrome) in 30% of the patients. Mean birth weight was 1728–2446 g and was lower in emergent CDs compared with planned CDs. Of note, compared to our research all these studies did not compare PAS pregnancies to a non-PAS group, had a relatively smaller amount of patients and described a relatively smaller amount of adverse outcomes. Fetal pH levels at birth were not described in any of these previous studies. These studies mainly showed high rates of NICU admissions and mechanical ventilation in PAS pregnancies. Apgar scores were inconsistent, while in our study Apgar score < 7 was significantly lower in PAS pregnancies. Gestational age at birth and birthweight were earlier and lower compared to our study’s results.

We found higher birthweights in the PAS group compared with the non-PAS group. Similarly, Jauniaux et. al found that fetal growth was not impaired when comparing pregnancies complicated by placenta previa with PAS (82 pregnancies) and with pregnancies without PAS (209 pregnancies) [26]. Nevertheless, other studies did demonstrate that preterm birth and small for gestational age infants appear to be more common in pregnancies complicated by PAS [27–29]. The abnormal fetal growth in pregnancies complicated by PAS may result from pathological implantation of the placenta that interferes with normal placental function. Gielchinsky et al. [27] compared 310 PAS pregnancies to non-PAS control group and found significantly more preterm deliveries and small for gestational age neonates in the PAS group, concluding that these findings may arise from pathological implantation of the placenta, resulting in interference with normal fetal growth.

Adverse neonatal outcome in PAS pregnancies, after adjustment for possible confounders, were independently positively associated with Apgar scores below 7 at 5 min, NICU admissions and composite adverse neonatal outcome. Possible reasons for the worse neonatal outcome in PAS pregnancies compared to non-PAS pregnancies may be attributed to the complexity of CD in a PAS pregnancy, as reported in previous studies, including higher rates of maternal bleeding and maternal blood transfusion [16]. The uterine incision in CD in a PAS pregnancy sometimes includes entry through the placenta before extracting the fetus. The abnormal placentation associated with PAS, as previously described [27], may allow less blood flow and thus lower reserves for the fetus in the minutes from beginning of surgery to the evacuation, thus rendering the fetus to worse outcomes. However, the exact reasons for this outcomes are rather intriguing and prompt further studies and research to understand them and hopefully manage to advise how to avoid them.

This study is not without limitations. Its retrospective design potentially introduces biases inherent to this type of study such as information bias due to different potential confounders. It is a single-center study, thus limited by the risk of bias due to individual clinician decisions regarding patient treatment. Since our tertiary medical center is a regional referral center for PAS pregnancies, the cohort presented in this study may be exposed to referral bias and might not be applicable and generalizable to other medical centers. Furthermore, the possibility of bias due to lack of adjustment for all possible confounders cannot be ruled out.

The main strength of this study is the inclusion of a relatively high number of PAS pregnancies matched to a larger cohort of non-PAS pregnancies describing the outcome of many neonatal characteristics never described before. Matching was performed for gestational age at CD and the number of prior CDs, thus limiting the possibility of selection bias. All PAS pregnancies included in our study were prenatally diagnosed and confirmed clinically or histologically.

Further prospective research including larger populations is required to further ratify these results and better inform and consult women in risk for PAS regarding their infants possible adverse outcomes.

## Conclusion

Pregnancies complicated by PAS are associated with adverse neonatal outcomes. This study demonstrated that neonates in PAS pregnancies had a higher proportion of Apgar score < 7 at 5 min, NICU admission and composite adverse neonatal outcomes compared with controls. Further studies are needed to underline the association of PAS and these adverse neonatal outcomes.

### Supplementary Information

Below is the link to the electronic supplementary material.

Supplementary file1 (DOCX 15 KB)

## Data Availability

Not applicable.
