# Supplementary material for: Neonatal outcomes in pregnancies complicated by placenta accreta- a matched cohort study
Source: Arch Gynecol Obstet. 2024 Jan 23;310(1):269–75. doi: 10.1007/s00404-023-07353-6 (PMC11169059; doi:10.1007/s00404-023-07353-6)
Supplement: Supplementary file 1 — Supplementary file1 (DOCX 15 KB) [file 404_2023_7353_MOESM1_ESM.docx]

**Table S1**. Indications for cesarean delivery in pregnancies without placenta accreta.

| Characteristic | No placenta accreta spectrum (n=1382) |
| --- | --- |
| Intrauterine growth restriction | 104 (7.5) |
| Suspected placental abruption | 75 (5.4) |
| Non-reassuring fetal heart rate | 100 (7.2) |
| Abnormal presentation | 54 (3.9) |
| Suspected macrosomia | 5 (0.4) |
| Placenta previa | 24 (1.7) |
| Previous cesarean delivery | 962 (69.6) |
| Suspected uterine rupture | 11 (0.8) |
| Suspected vasa previa | 8 (0.6) |
| Other indication | 39 (2.8) |

Data are given as median [Interquartile range] or n (%)
